# Multi-omic analyses in Abyssinian cats with primary renal amyloid deposits

**DOI:** 10.1038/s41598-021-87168-0

**Published:** 2021-04-16

**Authors:** Francesca Genova, Simona Nonnis, Elisa Maffioli, Gabriella Tedeschi, Maria Giuseppina Strillacci, Michela Carisetti, Giuseppe Sironi, Francesca Anna Cupaioli, Noemi Di Nanni, Alessandra Mezzelani, Ettore Mosca, Christopher R. Helps, Peter A. J. Leegwater, Laetitia Dorso, 99 Lives Consortium, Reuben M. Buckley, Danielle Aberdein, Paulo C. Alves, Asa Ohlsson Andersson, Gregory S. Barsh, Rebecca R. Bellone, Tomas F. Bergström, Adam R. Boyko, Jeffrey A. Brockman, Margret L. Casal, Marta G. Castelhano, Ottmar Distl, Nicholas H. Dodman, N. Matthew Ellinwood, Jonathan E. Fogle, Oliver P. Forman, Dorian J. Garrick, Edward I. Ginns, Bianca Haase, Jens Häggström, Robert J. Harvey, Daisuke Hasegawa, Isabel Hernandez, Marjo K. Hytönen, Maria Kaukonen, Christopher B. Kaelin, Tomoki Kosho, Emilie Leclerc, Teri L. Lear, Tosso Leeb, Ronald H. L. Li, Hannes Lohi, Mark A. Magnuson, Richard Malik, Shrinivasrao P. Mane, John S. Munday, William J. Murphy, Niels C. Pedersen, Simon M. Peterson-Jones, Max F. Rothschild, Clare Rusbridge, Beth Shapiro, Joshua A. Stern, William F. Swanson, Karen A. Terio, Rory J. Todhunter, Wesley C. Warren, Elizabeth A. Wilcox, Julia H. Wildschutte, Yoshihiko Yu, Leslie A. Lyons, Maria Longeri

**Affiliations:** 1 Department of Veterinary Medicine, University of Milan, 26900 Lodi, Italy; 2 Institute of Biomedical Technologies, National Research Council of Italy (CNR-ITB), 20090 Segrate, Italy; 3 Langford Vets, University of Bristol, Langford, BS40 5DU UK; 4 Department of Clinical Sciences of Companion Animals, Utrecht University, 3508 TD Utrecht, The Netherlands; 5 Pathology Service for Large Animals, Ecole Nationale Vétérinaire Oniris, 44300 Nantes, France; 6 Department of Veterinary Medicine and Surgery, College of Veterinary Medicine, University of Missouri, Columbia, MO 65211 USA; 7 School of Veterinary Science, Massey University, Palmerston North, 4442 New Zealand; 8 CIBIO/InBIO, Centro de Investigação em Biodiversidade e Recursos Genéticos/InBIO Associate Lab & Faculdade de Ciências, Universidade Do Porto, Campus e Vairão, 4485–661 Vila do Conde, Portugal; 9 Wildlife Biology Program, University of Montana, Missoula, MT 59812 USA; 10 Department of Clinical Sciences, Faculty of Veterinary Medicine and Animal Science, Swedish University of Agricultural Sciences, 750 07 Uppsala, Sweden; 11 HudsonAlpha Institute for Biotechnology, Huntsville, AL 35806 USA; 12 Department of Genetics, Stanford University, Stanford, CA 94305 USA; 13 Veterinary Genetics Laboratory, University of California - Davis, Davis, CA 95616 USA; 14 Department of Population Health and Reproduction, University of California - Davis, Davis, CA 95616 USA; 15 Department of Animal Breeding and Genetics, Swedish University of Agricultural Sciences, 750 07 Uppsala, Sweden; 16 Department of Biomedical Sciences, College of Veterinary Medicine, Cornell University, Ithaca, NY 14853 USA; 17 Hill’s Pet Nutrition Inc, Topeka, KS 66601 USA; 18 Reproduction, and Pediatrics, School of Veterinary Medicine, University of Pennsylvania, Philadelphia, PA 19104 USA; 19 Department of Clinical Sciences, College of Veterinary Medicine, Cornell University, Ithaca, NY 14853 USA; 20 Institute for Animal Breeding and Genetics, University of Veterinary Medicine Hannover, 30559 Hannover, Germany; 21 Department of Clinical Sciences, Cummings School of Veterinary Medicine, Tufts University, Grafton, MA 01536 USA; 22 National MPS Society, PO Box 14696, Durham, NC 27709 USA; 23 Department of Population Health and Pathobiology, College of Veterinary Medicine, North Carolina State University, Raleigh, NC 27607 USA; 24 WALTHAM Centre for Pet Nutrition, Freeby Lane, Waltham on the Wolds, Leicestershire, LE14 4RT UK; 25 AL Rae Centre of Genetics and Breeding, Massey University, Palmerston North, 4442 New Zealand; 26 Department of Psychiatry, University of Massachusetts Medical School, Worcester, MA 01655 USA; 27 Sydney School of Veterinary Science, Faculty of Science, University of Sydney, Sydney, NSW 2006 Australia; 28 School of Health and Behavioural Sciences, University of the Sunshine Coast, Sippy Downs, QLD 4558 Australia; 29 Laboratory of Veterinary Radiology, Nippon Veterinary and Life Science University, Musashino, Tokyo 180-8602 Japan; 30 Pediatrics and Medical Genetics Service, College of Veterinary Medicine, Cornell University, Ithaca, NY 14853 USA; 31 Department of Veterinary Biosciences; Department of Medical and Clinical Genetics, University of Helsinki and Folkhälsan Research Center, 00014 Helsinki, Finland; 32 Department of Medical Genetics, Shinshu University School of Medicine, Matsumoto, Nagano, 390-8621 Japan; 33 Symrise Group, SPF, Diana Pet food, 56250 Elven, France; 34 Department of Veterinary Science, University of Kentucky - Lexington, Lexington, KY 40506 USA; 35 Vetsuisse Faculty, Institute of Genetics, University of Bern, 3001 Bern, Switzerland; 36 Department of Surgical and Radiological Sciences, School of Veterinary Medicine, University of California Davis, One Shields Ave, Davis, CA 95616 USA; 37 Departments of Molecular Physiology and Biophysics, Cell and Developmental Biology, and Medicine, School of Medicine, Vanderbilt University, Nashville, TN 37232 USA; 38 Centre for Veterinary Education, University of Sydney, Sydney, NSW 2006 Australia; 39 Elanco Animal Health, Greenfield, IN 46140 USA; 40 Department of Veterinary Integrative Biosciences, College of Veterinary Medicine, Texas A&M University, College Station, TX 77845 USA; 41 Department of Medicine and Epidemiology, School of Veterinary Medicine, University of California Davis, Davis, CA 95616 USA; 42 Small Animal Clinical Sciences, College of Veterinary Medicine, Michigan State University, East Lansing, MI 48824 USA; 43 Department of Animal Science, College of Agriculture and Life Sciences, Iowa State University, Ames, IA 50011 USA; 44 School of Veterinary Medicine, Faculty of Health & Medical Sciences, University of Surrey, Guildford, GU2 7AL Surrey UK; 45 Department of Ecology and Evolutionary Biology, University of California Santa Cruz, Santa Cruz, CA 95064 USA; 46 Center for Conservation and Research of Endangered Wildlife (CREW), Cincinnati Zoo & Botanical Garden, Cincinnati, OH 45220 USA; 47 Zoological Pathology Program, University of Illinois, Brookfield, IL 60513 USA; 48 Division of Animal Sciences, College of Agriculture, Food and Natural Resources, School of Medicine, University of Missouri, Columbia, MO 65211 USA; 49 Department of Biological Sciences, Bowling Green State University, Bowling Green, OH 43403 USA

**Keywords:** Data integration, Proteomics, Kidney diseases, Genomics, miRNAs

## Abstract

The amyloidoses constitute a group of diseases occurring in humans and animals that are characterized by abnormal deposits of aggregated proteins in organs, affecting their structure and function. In the Abyssinian cat breed, a familial form of renal amyloidosis has been described. In this study, multi-omics analyses were applied and integrated to explore some aspects of the unknown pathogenetic processes in cats. Whole-genome sequences of two affected Abyssinians and 195 controls of other breeds (part of the 99 Lives initiative) were screened to prioritize potential disease-associated variants. Proteome and miRNAome from formalin-fixed paraffin-embedded kidney specimens of fully necropsied Abyssinian cats, three affected and three non-amyloidosis-affected were characterized. While the trigger of the disorder remains unclear, overall, (i) 35,960 genomic variants were detected; (ii) 215 and 56 proteins were identified as exclusive or overexpressed in the affected and control kidneys, respectively; (iii) 60 miRNAs were differentially expressed, 20 of which are newly described. With omics data integration, the general conclusions are: (i) the familial amyloid renal form in Abyssinians is not a simple monogenic trait; (ii) amyloid deposition is not triggered by mutated amyloidogenic proteins but is a mix of proteins codified by wild-type genes; (iii) the form is biochemically classifiable as AA amyloidosis.

## Introduction

The amyloidoses are a heterogeneous group of diseases widespread in humans and animals and are characterized by the abnormal deposition of aggregated and misfolded proteins, named amyloid, mainly in the extracellular space of various organs and tissues. Amyloid proteins are characterized by a high degree of β-pleated sheet secondary structure, forming insoluble fibrillary masses that are resistant to proteolysis^1^. The diagnosis of amyloidosis is based on amyloid detection by histology (via biopsy or necropsy). Amyloid deposits are staining positive with Congo red and show typical birefringence on polarization microscopy. The chemical identity of the prevalent protein in the deposits gives the name to amyloidosis^2^.

In humans, 36 different extracellular fibril protein types have been identified and related to different disease types. The well-known types are: AL amyloidosis, characterized by the deposition of the immunoglobulin light chains; ATTR amyloidosis, characterized by both wild type and mutated misfolded transthyretin proteins (TTR)^3^; and AA amyloidosis, characterized by the deposition of circulating serum amyloid A (SAA) as fibrillar protein (AA) in tissues^2^. Moreover, variations in genes leading to mutated proteins such as fibrinogen α, apolipoprotein A-I (ApoA-I), apolipoprotein A-II, apolipoprotein A-IV (ApoA-IV), and lysozyme can lead to hereditary systemic forms^2^. Also, the formation of the β-amyloid peptide in Alzheimer's Disease (AD) is widely recognized and investigated^4^.

In animals, systemic amyloidosis is rare and mainly characterized by a preponderance of protein AA in the deposits. Cases are reported as secondary to chronic inflammatory, infectious or neoplastic diseases in domestic animals, such as bovine^5^ and ovine^6^, wild mammals (*Gazella dorcas*^7^, *Panthera tigris altaica*^8^, *Panthera leo*^9^, *Mustela nigripes*^10^), and birds^11,12^. Cases of probable AL have been recorded in horses [13; 14], cats, and dogs, which are associated with plasmacytomas^15^. In non-humans, evidence of genetic predisposition to develop AA amyloidosis has been reported in the Shar-Pei dog breed^16,17^, in captive cheetahs (*Acinoynx jubatus*^18^), black-footed cats (*Felis nigripes*^19^), and domestic cat (*Felis catus*).

The domestic cat is reported to spontaneously develop amyloidosis at a young age and often without evidence of a pre-existent inflammatory condition. The disease has been described as primary and familial in two breeds: the Abyssinian, with a mainly renal form^20,21^, and the Siamese with a mainly hepatic form^22,23^. The three consistently reported features in Abyssinian renal amyloidosis are (i) the occurrence in related cats, (ii) the systemic deposition of AA protein with a significantly higher presence in kidneys, and (iii) the early death from renal failure. The trait is described as autosomal, and the influence of sex on the onset or development of the disease has not been reported in cats. The prevalence of the disease in any cat breed is unknown. Despite the “familial” transmission^23^, the mode of inheritance remained undetermined^23,24^. In a pioneering study^25^, the pre-eminent peptide in the amyloid renal deposits of an affected Abyssinian cat with a diagnosis of AA amyloidosis was sequenced. The complete primary structure of the SAA protein showed the amino-terminal residues were heterogeneous among different species of mammals, indicating a high tolerance for mutations in this region^26^. The cDNA sequencing of the N-terminal region in affected Abyssinians, Siameses, and domestic shorthair cats^24–27^ revealed some non-synonymous substitutions, however association of any genomic variation with the disease was not established.

In the last decade, mass spectrometry (MS)-based methods for human amyloid typing have been developed. Unlike the immunohistochemistry approach, which requires a panel of precursor specific antibodies and substantial observer expertise, MS determines objective identification of the proteins, allowing characterization of the deposits and the predominant fibril type^28^. Several proteomic approaches are now considered new clinical standards for amyloid typing, either on formalin-fixed paraffin-embedded (FFPE) or unfixed frozen specimens. Macrodissection or laser microdissection techniques are used to identify amyloid fibril composition of the major amyloid types, such as ATTR, AL, AA^29^, as well as rarer amyloid types: hereditary gelsolin^30^, ApoA-I^31^ and ApoA-IV^32^.

Studies in humans also highlighted the potential role of miRNAs in the accumulation of amyloid fibrils^33,34^. In cats, miRNA profiling is a rather recent achievement. The kidney has been profiled per se^35^ and some healthy and affected tissues/organs have been profiled to underlie their miRNA differential expression^36,37^. A more detailed characterization of the kidney miRNAome in cats was achieved by sequencing a feline kidney cell line before and after infection with mink enteritis virus^38^ and, more recently, using normal feline tissues^39^.

On tissues from complete necropsied cats, the following analyses were performed: (i) the whole genome variation of two affected Abyssinians *versus* 195 not affected cats of different breeds, (ii) the proteome from FFPE kidney specimens of three affected and three non-affected Abyssinians, and (iii) the miRNAome from the same six FFPE specimens. Data of this multi-omics investigation were integrated to hypothesize potential molecular mechanisms underlying the renal familial amyloidosis of the Abyssinian cat.

## Methods

Eight pedigreed Abyssinian cats subjected to complete necropsy at University Services during the past decade were included in the study; their tissues were donated by consenting owners for research (Table 1). All subjects were enrolled and classified according to their pathology reports, including gross necropsy and histology. The status was categorized as “affected” when the cat died at five years of age or younger, with a pathology report of amyloidosis confirmed by renal tissue positivity to Congo red staining with and without 0.25% acidified potassium permanganate pre-treatment and excluding other previous or intercurrent conditions, including acute and chronic inflammatory processes. In veterinary pathology, treatment with potassium permanganate solution of Congo Red allows identifying amyloid AA because amyloid AA becomes discolored, loses its affinity with Congo Red, and loses birefringence properties^40^. The subjects classified as “healthy” were three cats older than five years at death and with a report excluding amyloidosis and other disorders affecting kidney or both acute and chronic inflammatory systemic processes (controls). The cats were not first-degree relatives, but all belonged to related genetic lines where amyloidosis frequently occurred. Age, gender, specimens, analyses, and amyloidosis affected organs for each cat are also reported in Table 1.

**Table 1:** Abyssinian samples used in the study. “Sample_ID”: Fcat# is the accession number at the whole genome sequence feline 99 Lives initiative, PN# is the protocol number at the Department of Veterinary Medicine, University of Milan. “Cat_ID” is the identification number used in the main text, Fig. 4 and Fig. 5. “Status” is the health status related to amyloidosis. “Age at death” is reported as years, months. “Gender”: M = Male, F = Female, NF = Neutered Female. “Type” is the specimen DNA has been extracted from. “Analysis” are the omic analyses the sample has been subjected to. “Amyloid affected tissues” are the tissues with amyloid deposits at necropsy.

Cat ID	Sample ID	Status	Age at death	Gender	Type	Analysis	Amyloid affected tissues
A1	PN# 1/08 A	Affected	2	M	FFPE	Proteomics and miRNAs	Kidneys, spleen, lymph-nodes, gut associated lymphoid tissue, parathyroids
A2	PN# 160/09	Affected	5	F	FFPE	Proteomics and miRNAs	Kidneys
A3	PN# 233/02	Affected	4	NF	FFPE	Proteomics and miRNAs	Kidneys
A4	PN# 119/11	Healthy	5,8	NF	FFPE	Proteomics and miRNAs	= = =
A5	PN# 131/12	Healthy	6	F	FFPE	Proteomics and miRNAs	= = =
A6	PN# 44/14	Healthy	12	NF	FFPE	Proteomics and miRNAs	= = =
S1	Fact#18,778	Affected	4	F	Skeletal muscle	Genomics	Kidneys, Spleen, lymph-nodes, intestinal lamina propria, liver, thyroid and parathyroids
S2	Fact#16,515	Affected	6	F	Skeletal muscle	Genomics	Kidneys

Genomic DNAs from frozen skeletal muscles of two affected females (S1, S2) were whole-genome sequenced (WGS) at the University of Missouri in the context of the 99 Lives initiative and archived following the Institutional Animal Care and Use Committee protocol study protocols 9056, 9178, and 9642.

Formalin-Fixed Paraffin-Embedded (FFPE) kidney specimens of six cats, three with amyloidosis (one male, one female, one neutered female; A1, A2, A3 respectively) and three healthy (non-amyloidosis affected: one female, two neutered females; A5, A4, A6 respectively), were available at the Department of Veterinary Medicine, University of Milan and were analyzed individually for both proteomics and miRNAomics.

WGS data were produced as previously described^41^ and are accessible as part of the feline 99 Lives initiative (www.felinegenetics.missouri.edu/99Lives). Details of the whole genome sequencing, sequence processing, variant calling, and NCBI accession numbers have been previously reported^42^. The WGS data were aligned to the V9.0 *Felis catus* reference assembly^43^. WGS of 195 additional cats of different breeds and random-bred cats from the 99 Lives project (Control Population—Supplementary Table 1) were used for genomic variants detection. The Control Population included two other Abyssinians, one was the reference genome, and the other was enrolled in the 99 Lives Project for a different trait. All the WGS data were available via the 99 Lives project as Variant Call Format (VCF) file, retrieved from the *Felis catus* reference genome vs 9.0. The file included 84,005,772 variants.

The function “Activate Variants by Genotype Count Threshold” available through the SNP & Variation Suite v8.4 of the Golden Helix SVS software (Golden Helix, Inc., Bozeman, MT, www.goldenhelix.com) was used to detect the disease-associated variants. The VCF file was used as input, all the samples were set as “individual unrelated samples”, the two affected Abyssinians were set on “TRUE” and all the other cats were set on “FALSE” to be considered as controls. As the Control Population could theoretically include disease carriers, thresholds were set to consider only the variants present in both the affected cats, either with a heterozygous or homozygous genotype, and present in the remaining 195 cats with genotype frequency < 2% (counting both homozygous and heterozygous). The resulting variants were annotated and investigated for their potential function with the Ensembl Variant Effect Predictor (VEP), using the vs 9 of the cat genome assembly^44^. Missense, frameshift, and 3′UTR variants affecting the miRNA binding site were integrated with proteome and miRNAome data.

Proteome analysis was performed on kidney FFPE blocks. Two slices of 10 μm were cut from each block of the three affected and three healthy cats, Table 1. Specimens were mounted on glass slides and deparaffinized by incubating through two changes of xylene for 10 min each. The slices were rehydrated through a series of graded ethanol for 5 min each (100%, 95%, 70%), distilled water for 5 min, and then incubated in 50 mM Tris HCl buffer with mini protease inhibitors cocktail, for at least 30 min. Hydrated tissue slices were harvested with a scalpel blade and homogenized using a potter homogenizer in extraction buffer containing 8 M urea, 20 mM Hepes pH 8, with mini protease inhibitors cocktail. The homogenate was centrifuged (15 min, + 4 °C) to pellet non-homogenized tissue and cellular debris. The supernatant containing proteins were quantified with Bradford’s protein assay. Extracted proteins were subjected to reduction, alkylation, and protein digestion by using sequence-grade trypsin (Roche) overnight at 37 °C. The digestion was quenched by the addition of 2 μL of 98% formic acid, into the sample solution^45^. The proteolytic digest was desalted using a reversed-phase Zip-Tip C18 pipette tip (Millipore)^46^ and evaporated at 65 °C in a SpeedVac. Before Liquid Chromatograph Mass Spectrometer (LC–MS), samples were reconstituted in 0.1% formic acid and the Liquid Chromatography-electrospray Ionization-tandem Mass Spectrometry (LC–ESI–MS/MS) analysis was performed as published^47^.

MS spectra were searched against the *Felis catus* reference NCBI sequence database (release March 2019) by MaxQuant (version 1.6.0.1) using the following parameters: initial maximum allowed mass deviation of 15 ppm for monoisotopic precursor ions and 0.5 Da for MS/MS peaks, trypsin enzyme specificity, a maximum of two missed cleavages, carbamidomethyl cysteine as a fixed modification, N-terminal acetylation, and methionine oxidation, as variable modifications. False protein identification rate (1%) was estimated by searching MS/MS spectra against the corresponding reversed-sequence (Decoy) database. The minimum required peptide length was set to 6 amino acids and the minimum number of unique peptides supporting protein identification was set to 1. Quantification in MaxQuant was performed using label-free quantification algorithms (LFQ) based on the extracted ion intensity of precursor ions^47^. This extraction procedure does not distinguish between fibrillar and fibril associated soluble molecules, and this type of MS analysis cannot ascertain the locations of the specific proteins with confidence, because the aim was to characterize the proteome of the affected kidney.

Only proteins detectable and quantifiable in at least 2 out of 3 biological replicates were considered as positively identified in a sample and used for statistical analyses. Statistical analyses of Max Quant results were performed using the Perseus software module (version 1.4.0.8, www.biochem.mpg.de/mann/tools/) to identify proteins differentially expressed among the different conditions^48^. Proteins were considered differentially expressed if they were present only in one condition or showed a significant t-test difference (p-value ≤ 0.05).

Bioinformatic analyses were carried out by Panther software (Version 12.1)^49^ and DAVID software (release 6.8)^50^ to cluster enriched annotation groups of Cellular Component (GOCC), Biological Process (GOBP), Molecular Function (GOMF) and Pathways within the set of proteins up-regulated or present only in affected cats. The functional grouping was based on *p*-value ≤ 0.05 and at least three counts^51^. The mass spectrometry proteomics data have been deposited to the ProteomeXchange Consortium via the PRIDE^52^ partner repository with the dataset identifier PXD024140.

Kidney miRNAome analysis was performed on five sections of 10 μm sliced from the same six blocks used for the proteome analysis (Table 1). Total RNA was isolated using the miRNeasy FFPE Kit, with minor modification to the manufacturer instructions (Qiagen Handbook 06/2015). Briefly, after deparaffinization, the incubation to dewax tissue slices was increased up to 8 min, and gently shaking was applied. Samples were then eluted in 25 µl of RNase-free water. Isolated RNAs were quantified with the Qubit RNA HS (High Sensitivity) Assay Kits (#Q32855, Life Technologies) and their Quality Control (QC) was performed by Bioanalyzer Agilent RNA 6000 Nano Assay (#5067–1511, Agilent Technologies) in Agilent 2100 Bioanalyzer.

Approximately 200 ng of isolated RNA from individual kidneys was sequenced using the small RNA-seq kit by Illumina and the Illumina NextSeq500 platform, according to the Company’s protocol. The read length obtained was 1 × 75 bp for a total of 30 million reads per sample. The QC of the reads was assessed with FastQC (https://www.bioinformatics.babraham.ac.uk/projects/fastqc/) and the adapter sequences were removed using Cutadapt^53^. MiRNA mapping and profiling were obtained with miRDeep2^54^ and miRNAs were matched to the human homologs present in the miRbase database vs22.1^55^, as the cat miRNAome is still not available in the online databases.

MiRNAs with less than 10 reads per sample were removed from the dataset. Raw counts were normalized with the trimmed mean of the M-value method (TMM) to reduce errors and expressed as log2 counts per million. All the filtered miRNAs were used to evaluate the sample distributions through a multidimensional scaling (MDS) plot performed with R Studio routine (https://rstudio.com/products/rpackages/). Finally, the differential expression between miRNAs in affected *versus* control cats was assessed with a moderated t-test by limma, and nominal P-values were adjusted by the Benjamini–Hochberg method. For this analysis, only miRNAs having the genomic region supported by the highest number of reads were considered, and the significant miRNAs (P-value < 0.05) were represented using a heatmap. The heatmap was created with the R package "gplots", version 3.03 (https://rstudio.com/products/rpackages/).

MiRNA precursor sequences (pre-sequences) obtained with miRDeep2 that did not match any human miRNAs were aligned on miRbase database vs22.1 against *C. familiaris*, *B. taurus*, *S. scrofa, M. musculus,* and again *H. sapiens*. (BLASTN function).

For the identification of miRNA genes, the pre-sequences were aligned to the cat reference genome vs9.0 (BLAST tool of Ensembl genome browser 97). MiRNet database^56^ was used to predict if the identified Abyssinian miRNAs could target and regulate the proteins detected by proteomics. The prediction was made in human miRNAome since the cat was not available online, and the kidney was specified as the tissue in which the interactions take place. The identification of miRNAs binding sites on the mutated 3′UTR regions was performed through a customized algorithm retrieved from TargetScan vs7.2^57^.

## Results

### Genome variant detection

**Figure 1:**
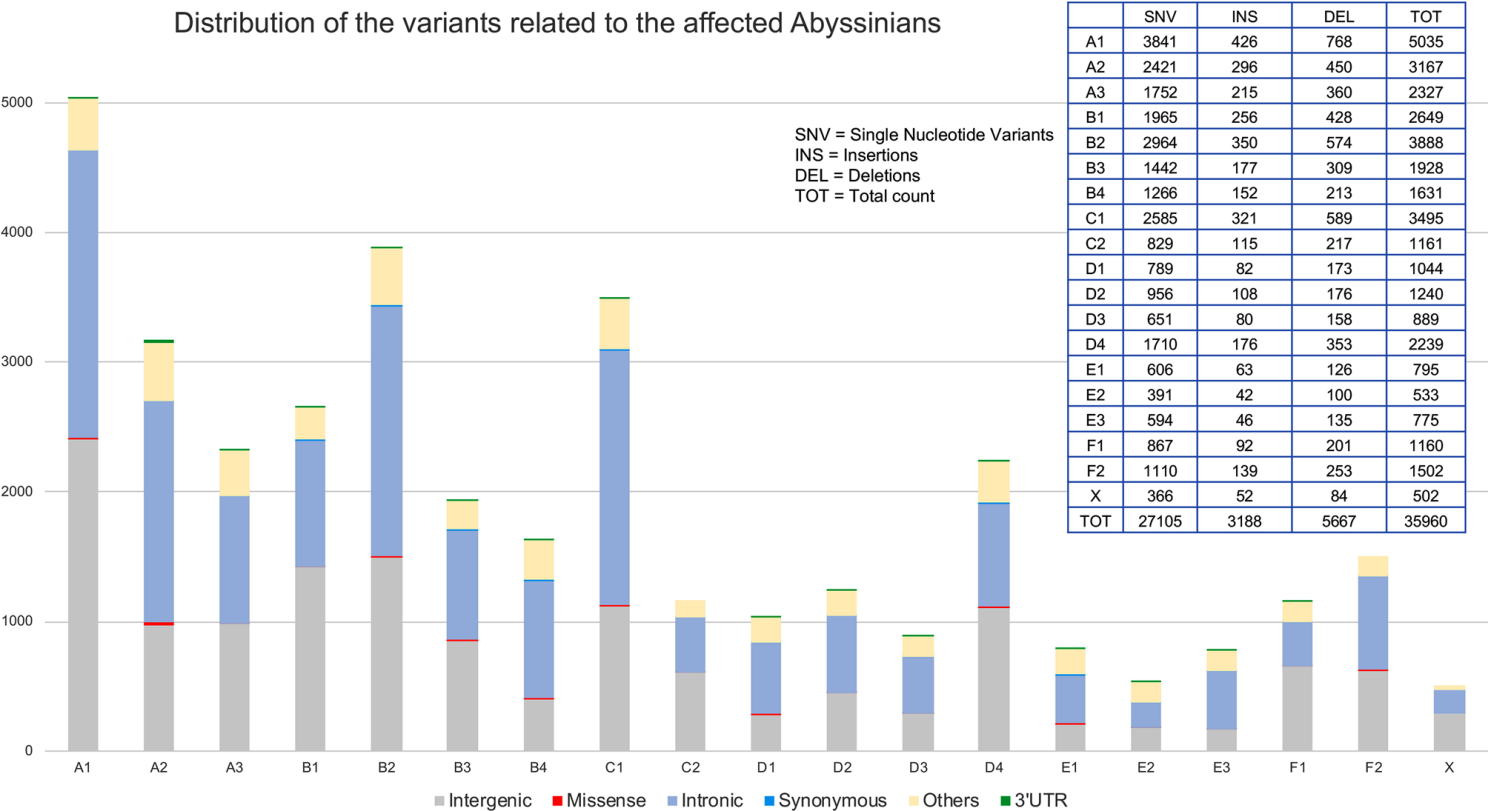
Overview of the different types of variants identified by Whole Genome Sequencing in the two affected Abyssinian cats compared to the Control Population with the Golden helix SVS Software. Variants distribution is reported for each cat chromosome. Variants referred to the class of “Other” included 5′UTR variants, downstream and upstream gene variants, non-coding transcript exon variants, and splice region variants.

Comparing the variants of the two affected Abyssinians to those of the 195 cats of the Control Population, 35,960 potential disease-associated variants were detected, the majority (31,045) mapped to intergenic or intronic regions (Fig. 1). The second most common variants (4623) were represented by the category of “Other” variants (Fig. 1) that included 5′UTR variants, downstream and upstream gene variants, non-coding transcript exon variants, and splice region variants. The 35,960 variants comprised 27,105 single nucleotide variants (SNV), 5667 deletions and 3188 insertions (Fig. 1). Considering the coding gene variants, 101 missense variants and one frameshift deletion were identified. Also, 84 variants mapping in 3′UTRs were detected and selected for additional analyses, given that SNVs in 3′UTR regions can modify miRNA binding sites, which can compromise gene expression^58^ (Supplementary Table 2). Overall, 186 variants were considered for additional analyses, in which 23 were homozygous and 113 heterozygous in both the affected cats and 50 were homozygous in one affected cat and heterozygous in the other (Supplementary Table 2).

### Kidney Proteome Analysis

Specimens, used for both proteome and miRNAome analyses (Table 1), came from the same six cats and in which both acute and chronic inflammatory processes were excluded with total necropsy and multi-organ histology. In affected cats, the kidneys were characterized by massive amyloid deposits. Focal to diffuse amyloid deposits had a typical salmon-pink color on Congo red staining in bright field and showed the characteristic apple-green birefringence under polarized light (Supplementary Fig. 1A, B). In the kidneys of the affected cats, amyloid deposition was found in interstitium, glomeruli, and blood vessels wall. Other organs of the affected cats with amyloid deposition are listed in Table 1.

Birefringence was not detected in potassium permanganate treated sections. Amyloid deposits were not detected in Congo red-stained sections of healthy cats observed both in bright field and under polarized light.

**Figure 2:**
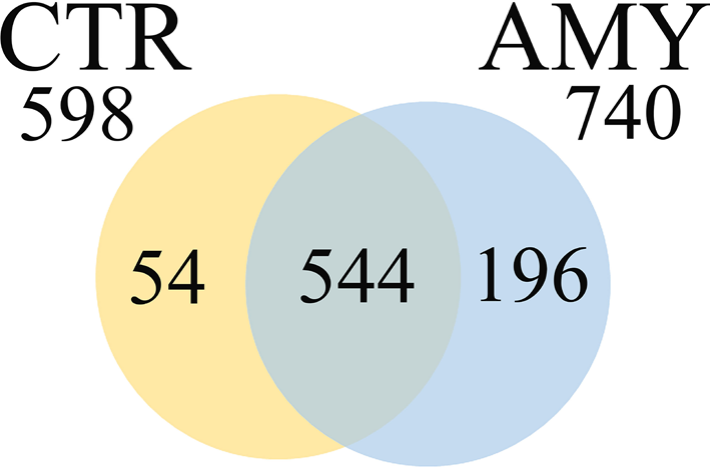
Venn diagram of the proteins identified in FFPE kidney tissues from cats affected by renal amyloidosis (AMY) and healthy (CTR). Only proteins present and quantified in at least 2 out of 3 repeats in each group were considered as positively identified.

**Figure 3:**
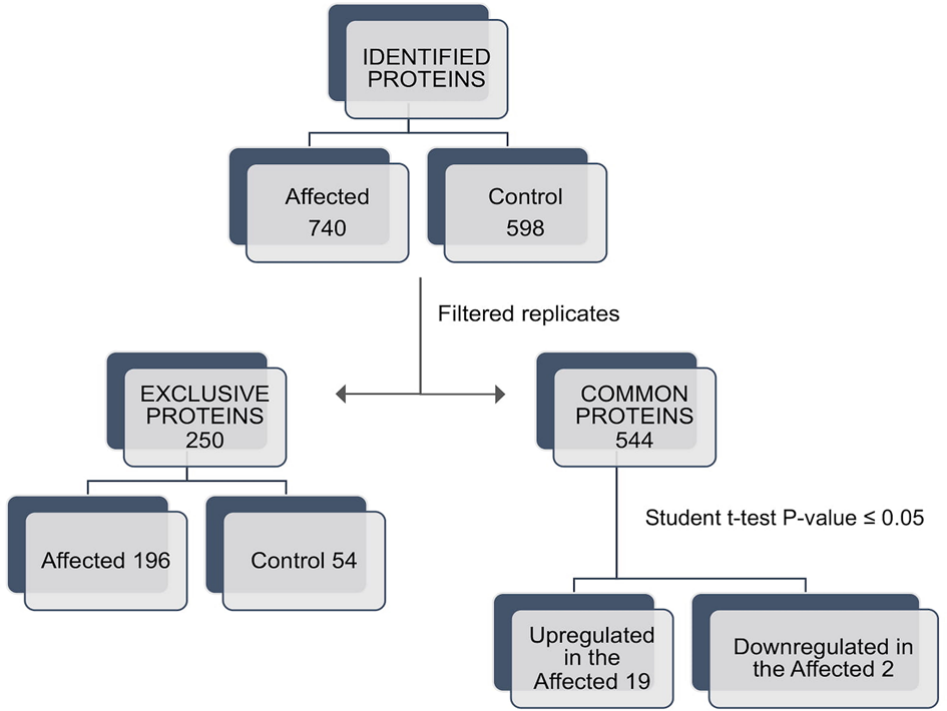
Workflow of the proteomic approach. A shotgun proteomic analysis was performed on FFPE kidney tissue from cats affected by renal amyloidosis (Affected) and healthy (Control). Statistical analyses were performed using the Perseus software (version 1.4.0.8, www.biochem.mpg.de/mann/tools/).

To characterize the protein component of the deposits, a quantitative label-free shotgun proteomic approach was adopted. The MS/MS data identify 740 proteins in kidneys with amyloid deposits and 598 proteins in the control kidneys. Proteins (n = 544) were expressed in both pathological and healthy tissues, while 196 proteins were exclusively present in the deposits and 54 proteins were expressed only in the controls (Fig. 2). Out of the 544 proteins common to the healthy and disease samples, 21 were differentially expressed (p-value ≤ 0.05, Student’s t-test): 19 proteins were up-regulated, and two proteins were down-regulated in cats suffering from amyloidosis (Fig. 3).

The exclusive and the differentially expressed proteins are listed in Supplementary Tables 3 and 4. Among proteins exclusively detected in pathological kidneys, SAA, ApoA-IV, and TTR were identified, with SAA being prevalent (Supplementary Table 3). By DAVID and Panther analyses, up-regulated and exclusively expressed protein in amyloidosis affected cats were related to main cellular components such as ribosome and extracellular matrix, as well as to cellular pathways involving oxidoreductase activity, the TCA cycle, and blood coagulation (Supplementary Table 5 and 6). These proteins were clustered in the following functional networks according to GO analysis (Supplementary Fig. 2: extracellular space region and matrix, metabolism, inhibition of serine protease activity and coagulation, antioxidant activity, impact on the structural constituent of ribosome, and translation.

### Kidney miRNAome Analysis

The quality of the total miRNA isolation was overall good and is shown in the length and quality plots of Supplementary Fig. 3. Weak peaks, corresponding to a small amount of other longer types of RNAs, were noticed for samples A4 and A5 that underwent a less efficient deparaffinising treatment (Supplementary Fig. 3).

For smallRNA-seq, the number of reads per sample ranged from 27,607,431 to 52,126,090 (mean value 40,267,032), with the highest number of reads per samples in the range of the 18–23 bp in length, which is in accordance with the standard 18-22 bp length of a mature miRNA sequence. Samples A1 and A4 showed more reads with 10–17 bp length, which could be addressed to the presence of non-miRNA short RNAs. Overall, a good sequencing quality was obtained (Supplementary Fig. 4). Sequencing data in fastq format were deposited in the ArrayExpress database at EMBL-EBI (www.ebi.ac.uk/arrayexpress/) under accession number E-MTAB- 8556.

The library size, the miRNA density, and the read count were evaluated before and after the normalization with the TMM method and are reported in Supplementary Fig. 5. A total of 854 different miRNA pre-sequences were identified with miRDeep2, corresponding to 618 unique mature sequences, reported with the same miRNA name (Supplementary Table 7). After filtering and normalization with miRDeep2, only 258 of 618 miRNA mature sequences were considered. The total read count for these miRNAs ranged from 7 reads (hsa-miR-4529-3p) to 29,052,681 reads (hsa-miR-10a-5p), with 58 miRNAs having less than 1,000 reads, 146 miRNAs ranging between 1,000 and 100,000 reads, and 45 miRNAs with more than 100,000 reads (Supplementary Table 7). Out of the 258 miRNAs, 71 had no match with human miRNAs according to miRDeep2, therefore the name assigned by the software (prefix “NC”) was retained. The remaining 187 were named according to the human nomenclature (prefix “hsa-mir”) (Supplementary Table 7). The manual BLAST of these 71 NC_miRNA pre-sequences showed eight new matches with humans that were not identified by miRDeep2 and were therefore added to the 187 “hsa-mir” for a total of 195 human miRNAs (Supplementary Table 8). Two miRNAs matched only to a bovine and a swine miRNA, while 61 miRNAs remained “NC” and therefore provisionally considered feline-specific miRNAs.

**Figure 4:**
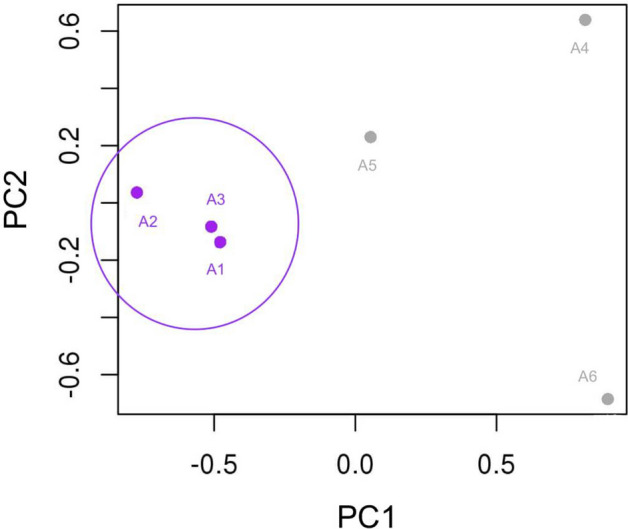
MDS plot realized with R Studio (https://www.rstudio.com/products/rpackages/), showing the cluster of the affected cats, based on the 258 miRNAs expression (log2 counts per million). Affected individuals (purple) are grouped on the left, while the healthy individuals are represented by grey dots on the right.

All the filtered miRNAs (n = 258) were used to evaluate the sample distribution with an MDS plot, and the affected cats clustered in a separate group (Fig. 4). The differential expression analysis showed 60 significant miRNAs (P-value < 0.05—Supplementary Table 9) with seven miRNAs significant also after the Benjamin-Hochberg correction (Adj. P-value < 0.05; Table 2).

**Table 2:** List of the seven miRNAs with significant differential expression in affected and control cats also after the Benjamini–Hochberg correction (Adjusted P-value < 0.05).

miRNA_ID (miRDeep2)	P-Value	Adj. P-Val	Read count
NC_018730.3_21281	0.0001	0.0189	69,021
NC_018727.3_13107	0.0002	0.0189	144
hsa-miR-4451	0.0002	0.0189	10,373
NC_018731.3_22438	0.0005	0.0318	192,694
NC_018726.3_8619	0.0007	0.0318	183
hsa-miR-4654	0.0007	0.0318	29
hsa-miR-186-5p	0.0011	0.0426	575,679

**Figure 5:**
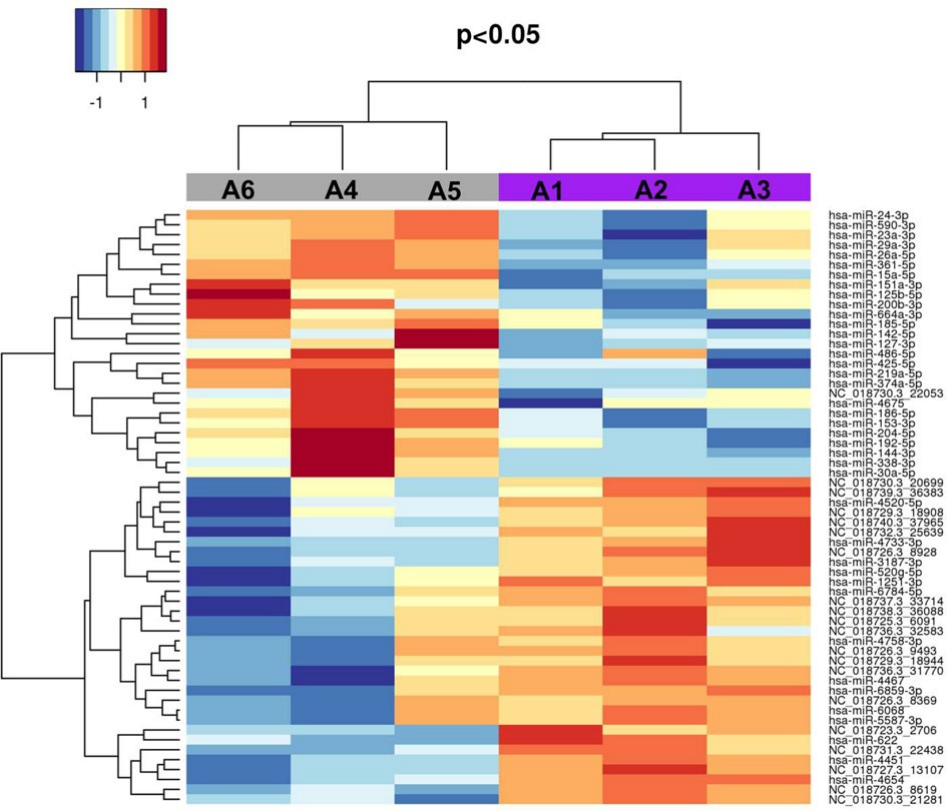
Heatmap created using the R package "gplots", version 3.03 (https://rstudio.com/products/rpackages/) with the 60 significant miRNAs (nominal P-val < 0.05). The grey bar and the purple bar represent the healthy (A4, A5, and A6) and the affected (A1, A2, A3) Abyssinians, respectively. The shades of blue refer to down-regulated miRNAs while the shades of red refer to up-regulated miRNAs.

The results shown by the MDS (Fig. 4) were confirmed with the heatmap based on the P-values of the 60 significant miRNAs. The plot showed four perfectly distinct blocks, with 27 miRNAs down-regulated in the affected samples and the remaining 33 miRNAs with the opposite trend (Fig. 5).

Out of the 60 miRNAs, 37 were already known in feline literature^38,39^ and three had human homologous. The remaining 20 miRNAs were considered new and exclusive to the cat (Supplementary Table 10). Twenty-one miRNA genes were already annotated, 37 aligned in non-annotated regions and one did not align to the cat reference genome V9.0^43^ (Supplementary Table 10).

### Omics integration

**Figure 6:**
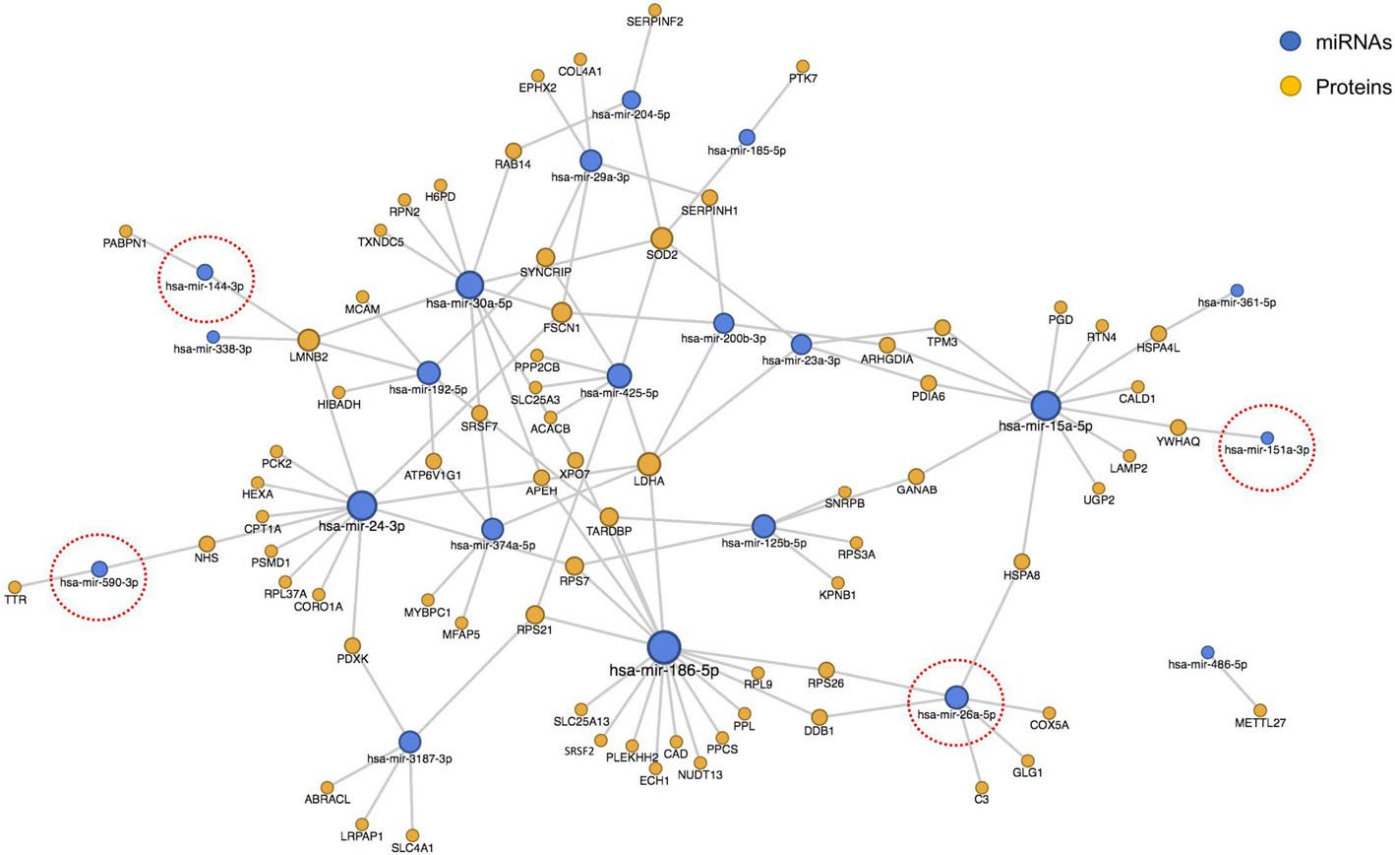
Network plot showing the interactions in pathological kidney tissue among the proteins exclusively present or up-regulated in the affected specimens and the miRNAs identified in the study, according to miRNet database. MiRNAs circled in red represent the only four miRNAs regulating exclusively the proteins related to the affected cats and none of those found in the controls.

Considering their interactions, 89 proteins are suggested to have regulation by 22 miRNAs (Supplementary Table 11). Hsa-miR-186-5p, hsa-miR-15a-5p and hsa-miR-24-3p had the largest number of targets (n = 18). Almost all the proteins in the network were expressed exclusively in the affected tissues, with only 18 out of 89 proteins exclusively present in the healthy controls (Supplementary Table 11). A clearer overview of the interactions that exclusively characterized the affected kidneys excluded these 18 proteins (Fig. 6). While most of the miRNAs regulated both the proteins related to the affected and the control cats, four down-regulated miRNAs are suggested to interact only with proteins exclusive or up-regulated in the affected tissues (sa-miR-26a-5p, hsa-miR-144-3p, hsa-miR-151a-3p and hsa-miR-590-3p, red circles in Fig. 6).

Searching for miRNA targets within the 84 3′UTR regions harboring the variants found by WGS, 14 miRNAs had their binding site in the mutated 3′UTR of 15 different genes (Supplementary Table 12).

## Discussion

Amyloidosis samples from cats are rare specimens, as not many breeders will take the expense to confirm their cat’s diagnosis by biopsy or necropsy, thus the cats with an accurate diagnosis are limited. As in most species, AA amyloid is related to long-term inflammation, the criterion adopted for the enrollment was the absence of acute/chronic inflammatory or tumor lesions in fully necropsied cats. The diagnosis of amyloidosis was determined by the histological specimens applying the Congo red staining routine protocols. In veterinary medicine, this method is still the gold standard for the detection of amyloid, and its sensitivity to potassium permanganate pre-treatment was used to preliminary identify amyloid deposits based on the fact that amyloid AA affinity for Congo red is lost in tissue sections incubated in potassium permanganate solution.

The study was almost exclusively on females (five of six). An effect of sex on amyloid deposition has never been described in cats, even a total uniformity in sex may have helped to further reduce the background variability. Some observed differences could also have been related to age. The study design needed a difference of age between the affected (younger) and the controls (older). Age-matched non-amyloidosis controls might have added to the significance of the observations. But young cats carrying unknown amyloid predisposition genes without lesions detectable with the classical histology at a very early stage could have been wrongly enrolled in the healthy group. Age differences in organ transcriptomes in both healthy and affected (as described in TTR amyloidosis in mice^59^) might still have accounted for some of the findings. Proteins and miRNAs differentially detected in affected compared to healthy were related to the pathology, but they could also have been modulated by differences in age and other factors (diet, stress, etc.) which was impossible to control and manage because of the different provenances of the cats. All the cats enrolled belonged to closely related lines, segregating for the disease. Abyssinians are quite homogeneous genetically^60^. Therefore, it can be assumed that these cats shared a large part of their gene pool and this helped to reduce much of the variability not related to the disease.

### Genome

In the present analysis genes that are known to be mutated in human inherited amyloidosis forms, such as *APP*, and *TTR* had variants matching the reference sequence. A great part of the variants detected in the affected cats mapped to intronic or intergenic regions. Since the function of these variants is still quite unknown in cats, future studies will improve the understanding of these polymorphisms. Several missense variants in coding genes, almost unique to the cases compared to the controls, were detected. These results must be confirmed on a larger number of samples, but they can be helpful to define a mutation profile of the amyloidosis affected Abyssinians. Two genes within those characterizing the affected cats (Supplementary Table 2) were reported in the literature as related to AA amyloidosis: *TREM1*  and *TNFRSF1A.*

*TREM1* encodes an immune receptor, belongs to the Immunoglobulin superfamily, and is expressed on mononuclear phagocytes. Soluble TREM1 levels were recently demonstrated to be significantly higher in patients with AA amyloidosis^61^. The mutations in the *TREM1* gene in the two affected Abyssinians could bring to the protein loss of function. Macrophages are instrumental in both accumulation and clearance of AA^61^, but *TREM1* mutation effect on amyloid deposition results unclear. Mutation in *TNFRSF1A* was recorded in one case of human AA secondary amyloidosis in kidney. The patient was a heterozygous mutant for two genes, including *TNFRSF1A*, developing an autoinflammatory syndrome characterized solely by amyloid deposits^62^.

The genomic analysis also showed some variants in the affected cats which mapped in genes directly involved in the development of diverse kidney syndromes. The Maltase—Glucoamylase gene (*MGAM*) had both one insertion and one deletion in the 3′UTR region, with both the variants being homozygous in the two affected cats (Supplementary Table 2). Despite no evidence supporting a correlation between *MGAM* and amyloidosis is reported in the literature, the protein codified by this gene has been identified as an important biomarker in acute kidney injury, due to its notably higher concentration in patients affected by this condition^63^. *FANCD2* and *FANCI Associated Nuclease 1* gene (*FAN1*) also had a missense variant homozygous in both the affected Abyssinians. Mutations in this gene resulted in chronic kidney disease^64^. According to the GeneCards database (https://www.genecards.org), other suggestive genes were *DAZ Interacting Zinc Finger Protein 1 Like (DZIP1L),* involved in the development of polycystic kidney disease, and *GREB1 Like Retinoic Acid Receptor Coactivator (GREB1L*), related to renal hypoplasia and/or renal agenesis.

These data could lead to considering that the onset of amyloidosis or the kidney major target in the Abyssinian cat can be facilitated by the presence of a compromised renal background.

### Proteome

The analysis performed by LC–MS/MS mass spectrometry showed that affected kidney tissues were characterized by well-known potentially amyloidogenic proteins as immunoglobulins, ApoA-IV, TTR, and SAA, the latter was prevalent. These findings are consistent with the data reported in the literature on kidney amyloid deposits, both in humans and cats. All the genes coding for these proteins had the reference alleles.

As shown in Supplementary Table 3, one of the most abundant proteins, exclusively identified in the affected tissues, was the constant region of the immunoglobulin G2 heavy chain. Although the presence of immunoglobulins heavy chains is not common in amyloidosis, rare instances of amyloid fibrils derived from the immunoglobulin heavy chain, designated heavy chain amyloidosis (AH), have been reported^65,66^. In this study, the abundant presence of this immunoglobulin cannot be easily attributed to an ongoing inflammation process, because of the criteria applied to the cat enrollment. Furthermore, the GO analysis conducted on the up-regulated and exclusively expressed proteins in affected cats did not show an enrichment of the pathway of the inflammatory immune response, excluding this scenario.

Interestingly, SAA was exclusively detected in the kidney of the affected cats. The *Felis catus* protein database contains several isoforms of whole or partial SAA protein, as also reported in Supplementary Table 3. The proteomic analysis identified three different isoforms of the SAA protein, two of which corresponding to partial sequences, according to the databank. The presence of abundant wild type AA in the deposits but not constant high SAA concentration, as experienced in the practice and reported in a longitudinal story on affected Abyssinians^67^, has suggestive similarities both with the suggested mechanism of rapid incorporation of wild type AA into the deposits drastically reducing the circulating SAA concentration, recorded in a mouse model^68^ and with the unresolved issue of a short seeding and nucleation that could make unnecessary chronic high SAA concentrations for docking and misfolding in the tissues^69^.

Besides, Supplementary Table 3 reports the presence of TTR protein, which, in humans can cause familial forms of amyloidosis, although, until now, there is no experimental evidence of TTR amyloidogenicity in cats.

The GO analysis clustered the up-regulated and exclusively expressed proteins in the affected Abyssinians into five distinct classes: extracellular space region and matrix (ECM), metabolism, inhibition of serine protease activity and coagulation, antioxidant activity and impact on the structural constituent of ribosome, and translation, (Supplementary Fig. 2, Supplementary Table 5, Supplementary Table 6), suggesting a mixed panorama of proteins representative of different phases and aspects of the progressive tissue damage occurring from the onset to the late disease. These results are also consistent with previous studies reporting that the protein composition of insoluble deposits in the extracellular spaces can include ECM components^70^ and suggesting that the ECM modulates the level of amyloidogenic peptides^71^. These effects could be related to direct regulation of the amyloidogenic protein expression by ECM signaling molecules and/or to modulation of protein processing by the ECM related cytoskeletal network in the cytoplasmic compartment^72^. Furthermore, the results of the present study showed that renal amyloidosis affects catabolic processes. This is consistent with previous findings suggesting that ATP, at concentrations of physiological significance to both intra and extracellular environments, actively participates in fibrillogenesis and is involved in a different form of amyloidosis^73^.

Those that can be considered as potential secondary effects were represented in the remaining GO categories. The present study reported an increase in categories related to serine-type endopeptidase inhibitor activity with significant enrichment in proteins involved in this process and the blood coagulation pathway. Bleeding manifestations is a multifactorial event, which can occur in human patients with an advanced stage of amyloidosis. In general, the vascular infiltration of amyloid fibrils can disrupt vascular integrity, and bleeding manifestations in patients with renal amyloidosis may be augmented by acquired hemostatic abnormalities associated with renal dysfunction^74^. Moreover, acquired deficiency of serine endopeptidase factor X (Stuart factor) is the most common coagulation factor deficiency that has been identified in patients with amyloidosis^75^, probably due to the apparent ability of amyloid fibrils to bind factors X^74^.

The increased oxidoreductase and antioxidant activity in affected kidneys (Supplementary Table 5, Supplementary Table 6) could be related to the progressive deposition of amyloid fibrils in organs. Indeed, increased levels of oxidative stress around the amyloid deposits have been detected in a variety of amyloid diseases^76^. However, it is still poorly understood whether oxidative stress is involved in the progression of amyloidosis.

Then again, Panther data analysis showed that renal amyloidosis in cats influenced both ribosomes and translation processes. These data agreed with previous studies demonstrating that in vivo protein aggregate accumulation was mainly composed of ribosomal components^77^.

### miRNAome

MiRNAs found in affected kidneys were used in gene target prediction study and the correspondence of miRNA target messengers and proteins founded were evaluated, giving a deeper insight into the cellular mechanisms of the disorder. The broad range of expression of miRNA analysed was already reported in the literature^57^ and it was shown to be related to the functional roles of miRNAs in a specific tissue or in a particular functional frame, which could influence their higher or lower expression.

Four significantly down-regulated miRNAs mainly characterized the interactions that took place exclusively in the pathological tissues (Fig. 6). They target two known “amyloidogenic” proteins (TTR and PABPN1) and proteins whose solubility was changed by the deposit’s presence^78^. Hsa-miR-144-3p and hsa-miR-590-3p regulated the expression of *TTR* and Poly(A) Binding Protein Nuclear 1 (*PABPN1*) respectively, with the first one reported as involved in the TTR amyloidosis deposits^58^ and the second one in the amyloid-like fibrils of the Oculopharyngeal Muscular Dystrophy^79^. Hsa-miR-26a-5p regulated six genes including the one coding for Complement C3 (C3), whose upregulation has been reported facilitating the deposit clearance^80^, and that coding for the Heat shock 70 kDa protein (HSPA8), which was one of the proteins demonstrated to lose their solubility following an extracellular accumulation of amyloid in a mouse model of Alzheimer-type amyloidosis^81^. YWHAQ was also among the proteins reported by Xu et al*.*^78^ and it was regulated by hsa-miR-151a-3p, the fourth down-regulated miRNA.

The proteins—miRNAs network showed, once again, different phases of the disease. The two nodes of the network belonging to hsa-miR-26a-5p and hsa-miR-151a-3p were representative of the cell response to the mechanisms activated by the deposition of amyloid plaques, while the nodes represented by hsa-miR-144-3p and hsa-miR-590-3p were suggestive of more direct potential triggers in the disease process.

The TargetScan database algorithm was used to predict which binding site of the differentially expressed miRNAs occurred in the mutated 3′UTR regions of the genes identified through the genomic analysis. Fifteen genes were mutated in a 3′UTR binding site.

Among these genes, two were the most interesting based on their functions. The variant in the 3′UTR region of the Matrix Metalloproteinase 1 (*MMP1*) gene occurs in the binding site of miR-374a-5p, which was among the down-regulated miRNAs. MMP1 is known for its role in the proteolysis of AA fibril proteins^82^. However, like the majority of the 3′UTR variants identified, the variant characterizing the 3′UTR of *MMP1* was a SNV and one single base mismatch could not always completely compromise the binding of the miRNA. On the other hand, an interesting result was noticed for the already mentioned *MGAM* gene, which was also characterized by missense variants. In this case, the 3′UTR variant had a consistent insertion of 22 bp, occurring in the binding site of miR-6784-5p.

The latter result was engaging considering the integration of all three omics. *MGAM* was the only gene common to the three different analyses. It had both one insertion and one deletion in the 3′UTR region, both homozygous in the two affected cats (Supplementary Table 2). In particular, the insertion in the 3′UTR of *MGAM* occurred in the binding site of miR-6784-5p, one of the up-regulated miRNAs in the affected. Moreover, the MGAM protein was among the proteins identified exclusively in the affected cats. Despite no evidence supporting a correlation between MGAM protein and amyloidosis was reported in the literature, it was identified as an important biomarker in acute kidney injury, due to its notably higher concentration in patients affected by this condition^63^. Therefore, the 3′UTR variant may cause the missed binding with miR-6784-5p and the consequent loss of the protein expression silencing, explaining the exclusive presence of MGAM protein in the affected tissues.

### Conclusions

The multi-omics data integration emphasized the presence of different genes involved in weakening and predisposing the kidney to the development of different syndromes, also creating a favorable background for the insurgence of amyloidosis and its side effects. Major limits of the present study were the low number of samples (due to their rarity), the difference in age (due to the features of the disease), the absence of a transcriptome analysis (impracticable on FFPE), and the tools used for some data analysis (essentially based on human references). Beyond that, the metadata available through this study can be an important hint for the reconstruction of the molecular basis of feline and other amyloidoses.

Overall, the present data demonstrated Abyssinian amyloidosis as polygenic with several low-penetrance genetic variants. This finding is consistent with a recent study where evidence for a complex genetic basis for the disease in Oriental shorthair cats was found^83^. The genomic results suggested a complex molecular background characterizing feline renal amyloidosis, with different mutated genes directly interacting with AA fibrils pathway, and other mutated genes more involved in the kidney impairment.

The biochemical characterization of the kidney deposits by mainly AA and some other well-known amyloidogenic proteins, such as TTR and ApoA-IV, confirmed what was reported in the classical feline literature and classifies the Abyssinian form within the “AA amyloidoses”^25^. This is consistent with the loss of affinity for Congo Red stain in potassium permanganate treated tissue sections of affected cats. All these classic amyloidogenic proteins resulted to be wild type in the genomic analysis. This might suggest their massive accumulation in the deposits as a secondary event.

Despite in a recent study on FFPEs of two unknown or mixed breed cats with systemic amyloidosis, proteome analysis showed positivity to SAA but also APOE and not ApoA-IV^84^, differences in breed and absence of healthy controls, information on age and sex make the comparison between the data of the two works incongruous.

The present work shows how a multi-omics approach can be useful for complex trait studies also in animals and be a source for further annotations and meta-analyses. A list of 102 missense variants and 84 3′UTR variants potentially related to Abyssinian feline amyloidosis is now available and can be used for a population screening in future studies to confirm possible associations. Proteomics gave further support in characterizing the depositions in the affected tissues, confirming already known amyloidogenic proteins and outlining a protein differential expression profile.

Finally, miRNAs differential expression profile was defined, and 20 novel feline miRNAs were identified.

## Data availability

All the six FFPE samples (PN#) were out of the archives at the Department of Veterinary Medicine—University of Milan—Organismo Preposto al Benessere degli Animali OPBA-56–2016 approval; all methods were carried out following relevant guidelines and regulations. All the genomics data that support the findings of this study are available through the 99Lives Initiative, are published in the NCBI SRA and the accession numbers are also available in the Supplementary material of Buckley, R.M. et al*.*^42^; at https://www.mdpi.com/2073-4425/11/6/682/s1. The samples S1 and S2 are NCBI biosample# SAMN05980344 and SAMN08924116. All the mass spectrometry proteomics data have been deposited to the ProteomeXchange Consortium via the PRIDE partner repository. The dataset identifier is PXD024140. All the miRNAs sequencing data in fastq format are available on the ArrayExpress database at EMBL-EBI (www.ebi.ac.uk/arrayexpress/), under the accession number E-MTAB- 8556.

## Supplementary Information

Supplementary Information 1.

Supplementary Information 2.

Supplementary Information 3.

Supplementary Information 4.

Supplementary Information 5.

Supplementary Information 6.

Supplementary Information 7.

Supplementary Legends.
