# Supplementary figures and images for: Multi-omic analyses in Abyssinian cats with primary renal amyloid deposits
Source: Sci Rep. 2021 Apr 16;11:8339. doi: 10.1038/s41598-021-87168-0 (PMC8052419; doi:10.1038/s41598-021-87168-0)

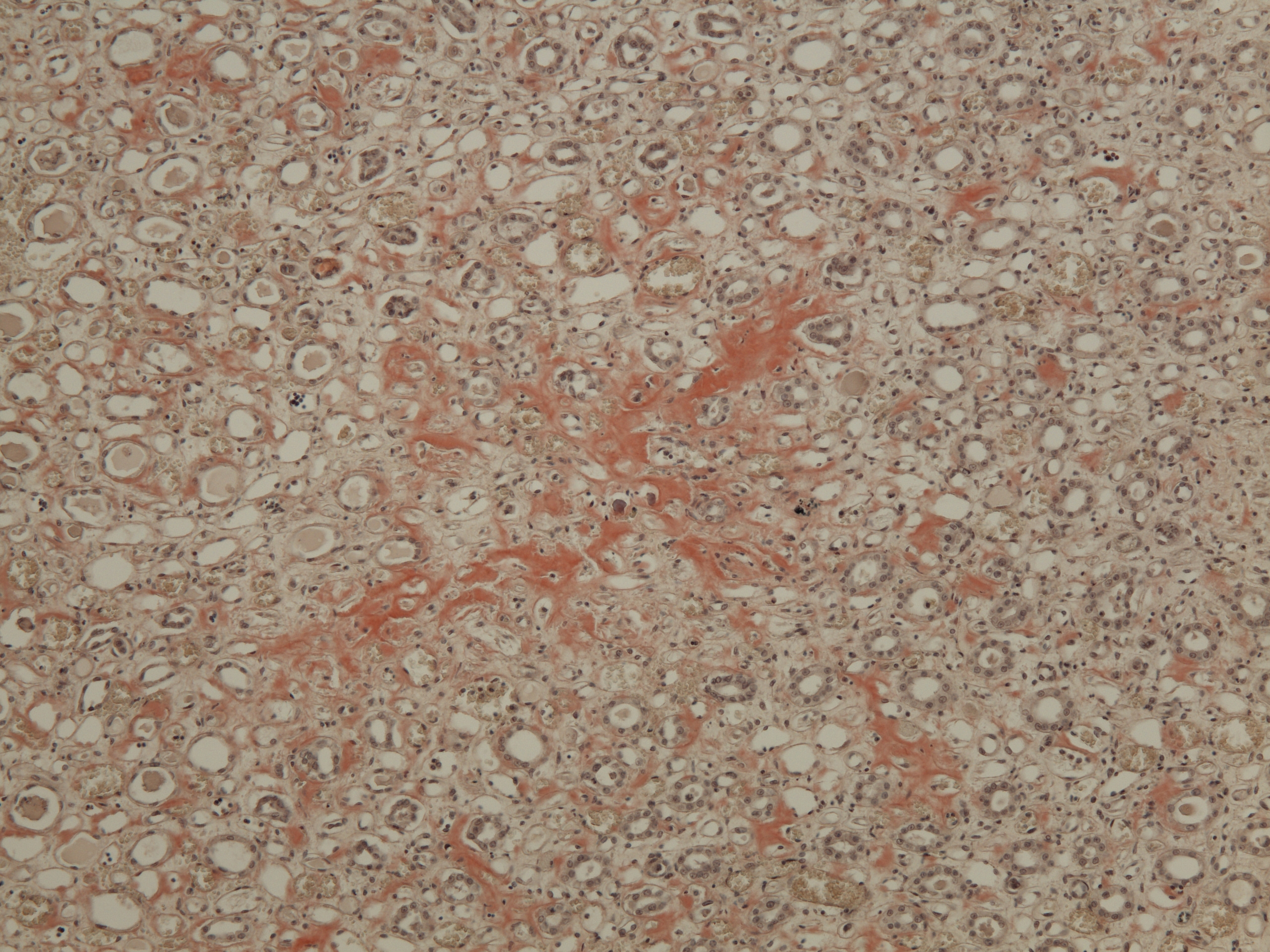

Supplement: Supplementary file 2 — Supplementary Information 2. [file 41598_2021_87168_MOESM2_ESM.jpg]

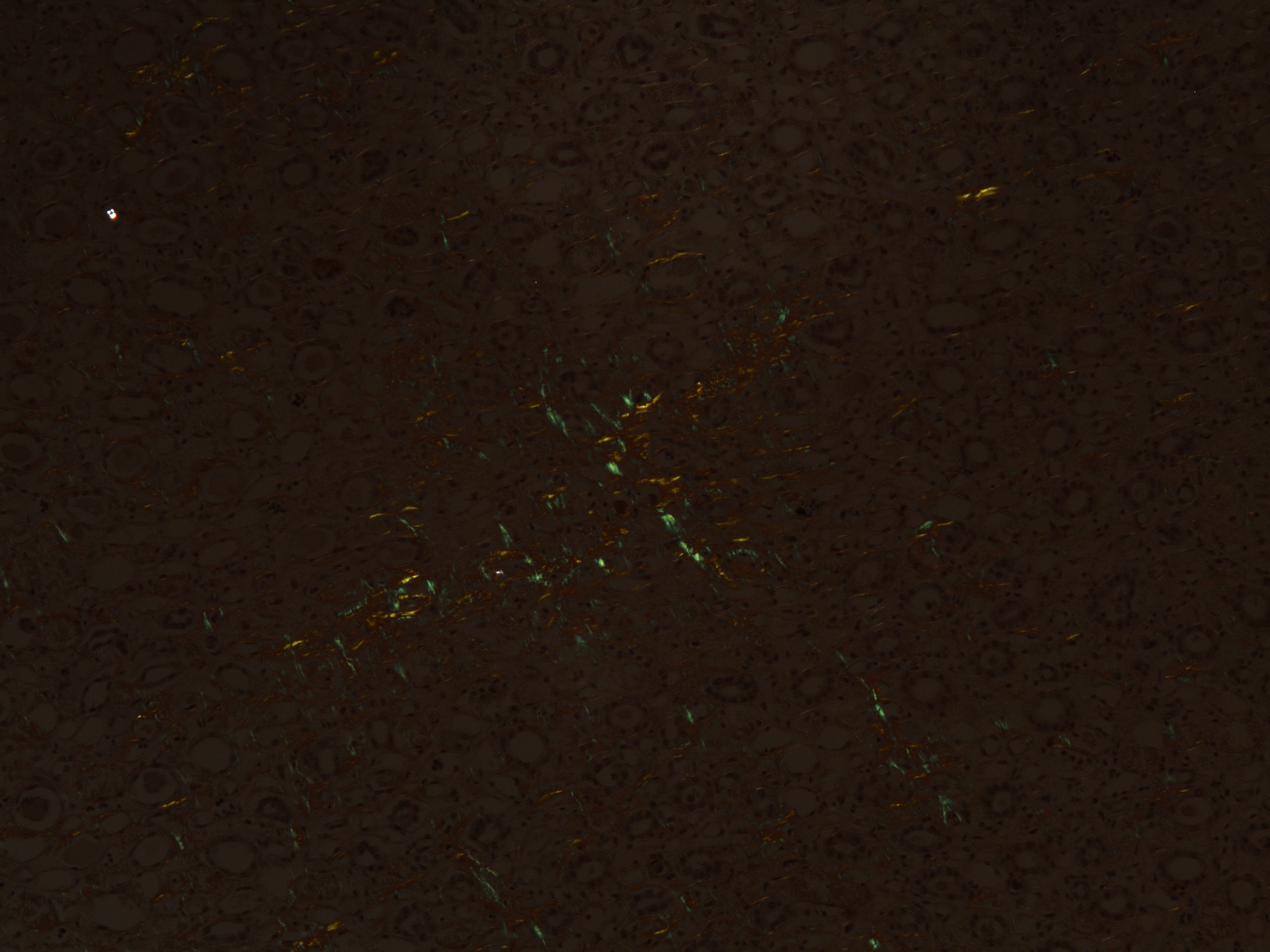

Supplement: Supplementary file 3 — Supplementary Information 3. [file 41598_2021_87168_MOESM3_ESM.jpg]

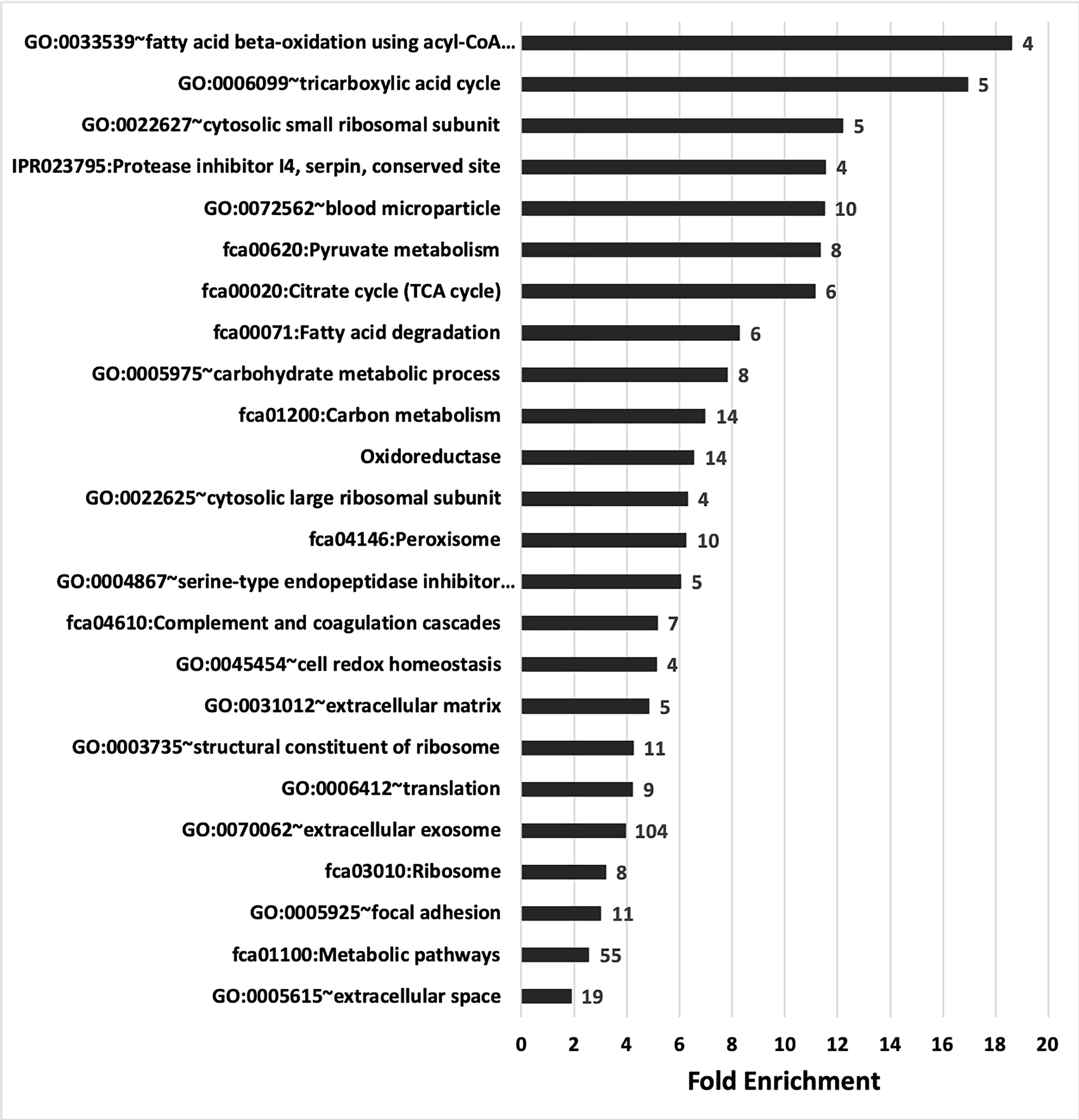

Supplement: Supplementary file 4 — Supplementary Information 4. [file 41598_2021_87168_MOESM4_ESM.jpg]

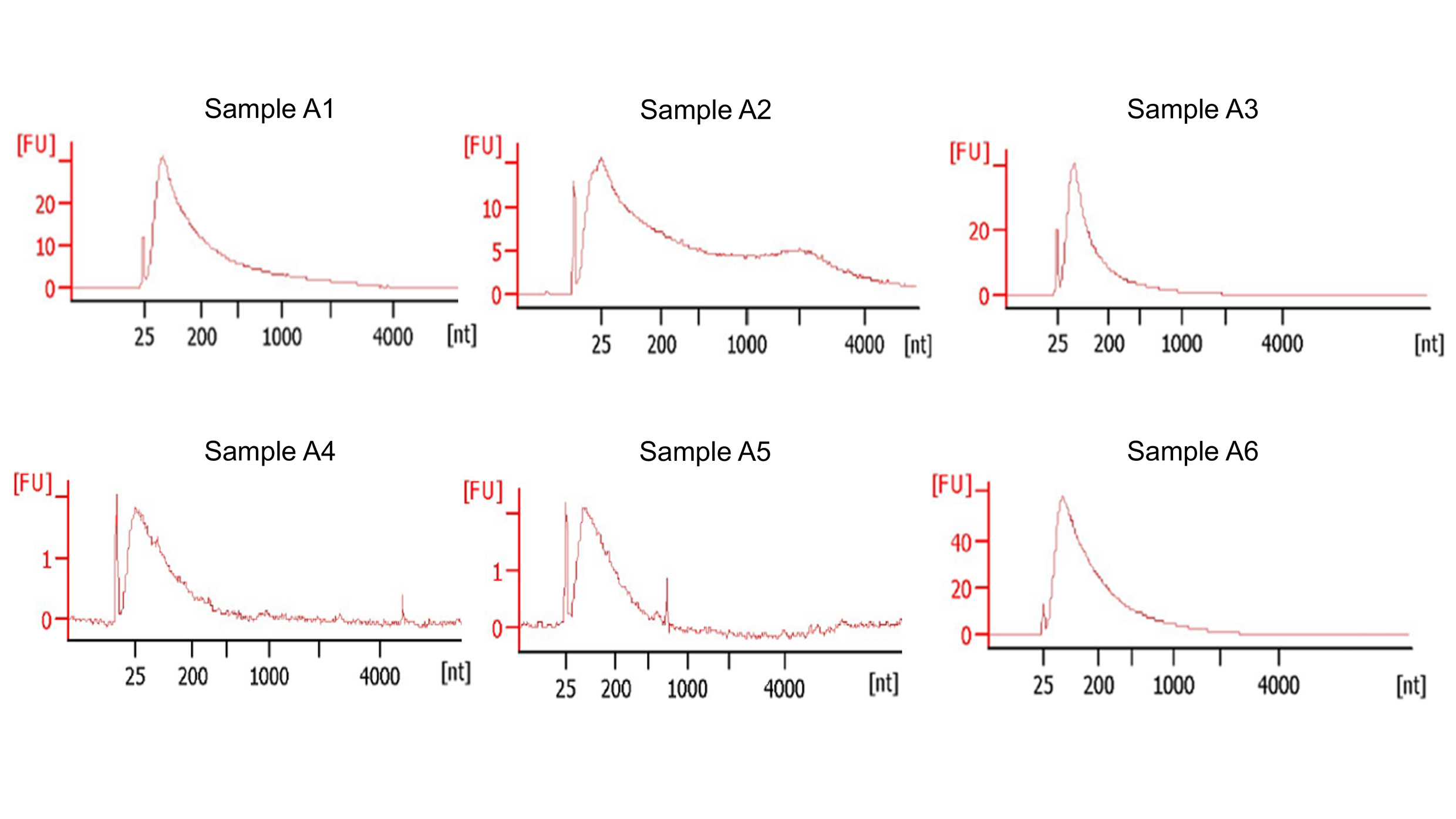

Supplement: Supplementary file 5 — Supplementary Information 5. [file 41598_2021_87168_MOESM5_ESM.jpg]

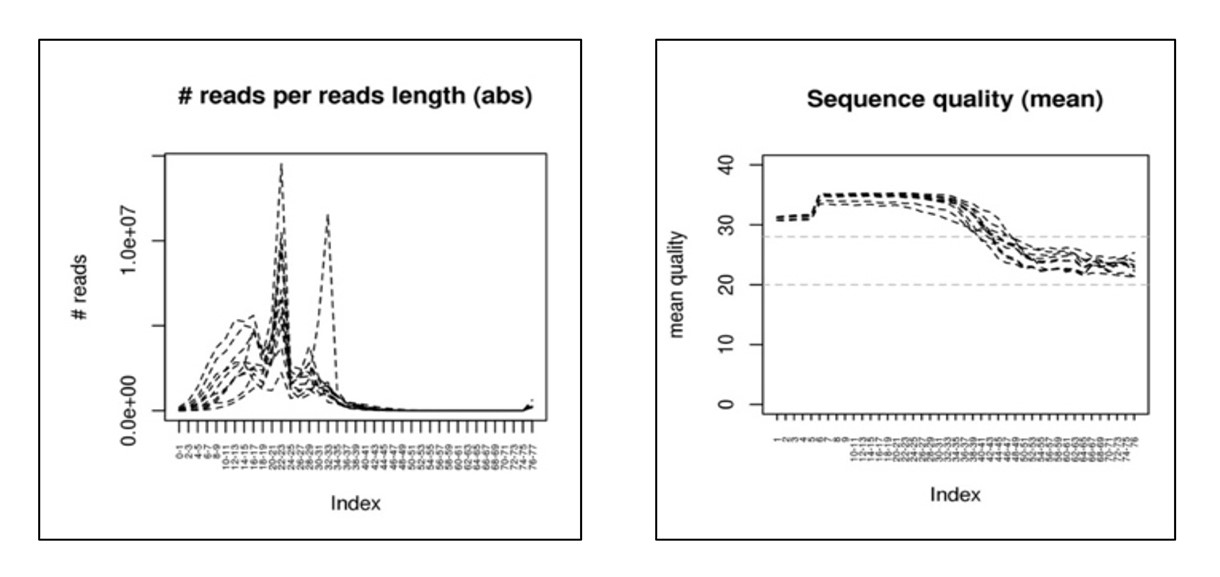

Supplement: Supplementary file 6 — Supplementary Information 6. [file 41598_2021_87168_MOESM6_ESM.jpg]

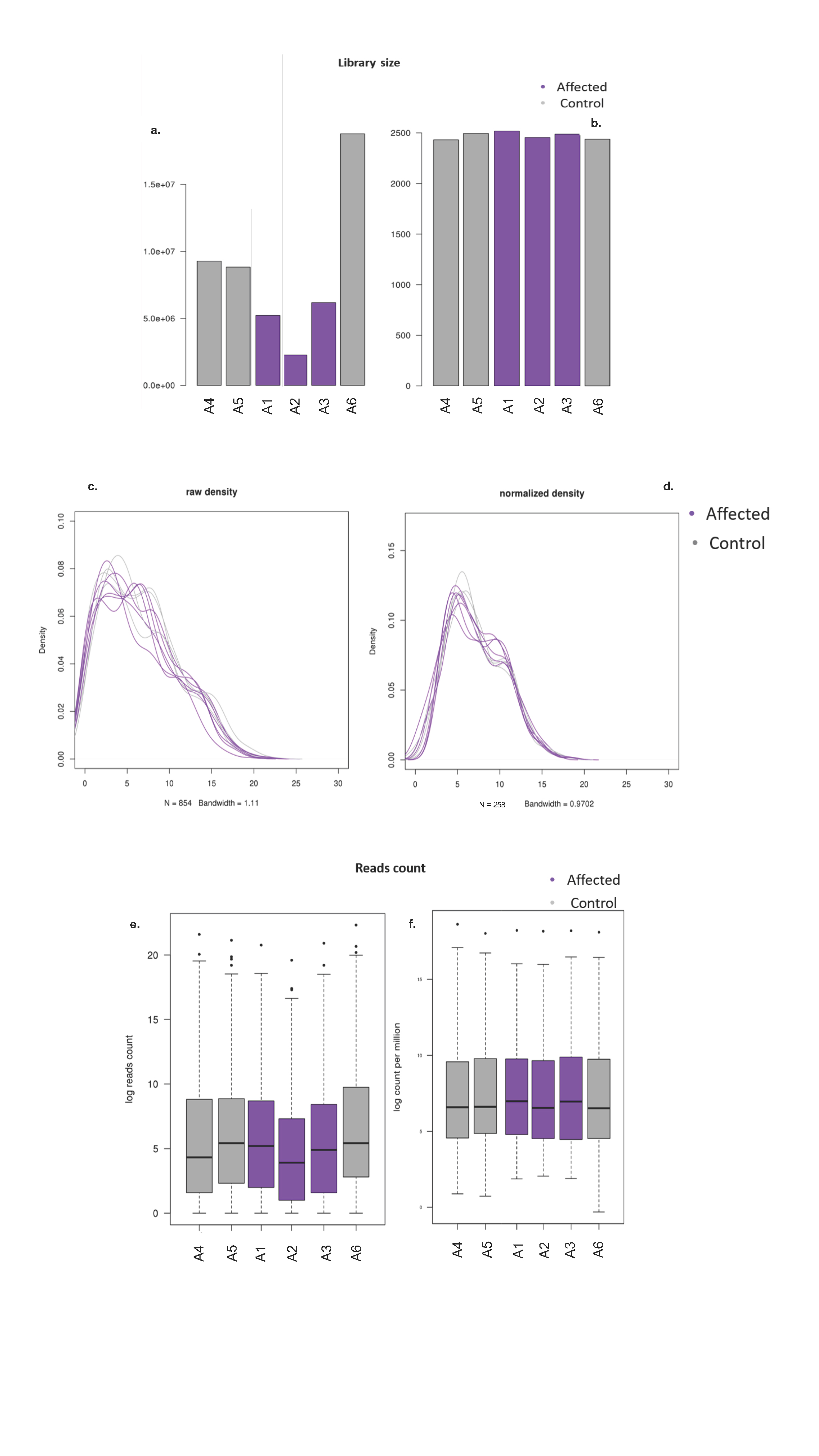

Supplement: Supplementary file 7 — Supplementary Information 7. [file 41598_2021_87168_MOESM7_ESM.jpg]
