# Supplementary material for: Multi-omic analyses in Abyssinian cats with primary renal amyloid deposits
Source: Sci Rep. 2021 Apr 16;11:8339. doi: 10.1038/s41598-021-87168-0 (PMC8052419; doi:10.1038/s41598-021-87168-0)
Supplement: Supplementary file 8 — Supplementary Legends. [file 41598_2021_87168_MOESM8_ESM.docx]

**SUPPLEMENTARY FIGURE LEGENDS**

**Supplementary Figure 1**. Affected cat, kidney section stained with Congo red and photographed in bright field (A) and under polarized light (B). Magnification 100 x. Interstitial amyloid deposits in the renal medulla are salmon-pink stained under non-polarized light and birefringent under polarized light. Staining and birefringence are absent in potassium permanganate pre-treated sections (data not shown).

**Supplementary Figure 2.** Distribution into the categorical annotation of the proteins up-regulated or present only in pathological kidneys. The bar chart shows the fold enrichment of the most enriched categories. Numbers next to the bars indicate the count.

**Supplementary Figure 3.** Bioanalyzer Agilent RNA 6000 Nano Assay plots of samples characterized by a double step of deparaffinization. It is possible to notice two well distinguished peaks: the first one on the left represents the marker, while the second peak corresponds to the amount of isolated miRNAs. Possible further peaks (sample A4; A5) can be referred to as potential contaminations of non-miRNA short RNAs.

**Supplementary Figure 4.** Quality control of the sequencing data with FastQC. The left image shows the number of reads for each read length. The right image reports the overall sequence quality.

**Supplementary Figure 5.** The figure shows the sequencing data before and after the normalization with the TMM method. On the left, figures **a, c,** and **e**, show the Library Size, the miRNA density, and the read count before the normalization. Figures **b, d,** and **f** represent the normalized sequencing data.
